# Early Changes in Ocular Surface and Tear Inflammatory Mediators after Small-Incision Lenticule Extraction and Femtosecond Laser-Assisted Laser In Situ Keratomileusis

**DOI:** 10.1371/journal.pone.0107370

**Published:** 2014-09-11

**Authors:** Shaohui Gao, Saiqun Li, Liangping Liu, Yong Wang, Hui Ding, Lili Li, Xingwu Zhong

**Affiliations:** 1 Zhongshan Ophthalmic Center and State Key Laboratory of Ophthalmology, Sun Yat-sen University, Guangzhou, China; 2 Henan Eye Hospital and Henan Eye Institute, People's Hospital of Zhengzhou University, Zhengzhou, Henan Province, China; 3 Hainan Eye Hospital, Zhongshan Ophthalmic Center, Sun Yat-sen University, Haikou, Hainan Province, China; Boston University School of Medicine, United States of America

## Abstract

**Purpose:**

To characterize the early ocular-surface changes or tear inflammatory-mediators levels following small-incision lenticule extraction (ReLEx smile) and femtosecond laser-assisted laser in situ keratomileusis (FS-LASIK).

**Methods:**

Forty-seven myopic subjects were recruited for this prospective study. Fifteen underwent ReLEx smile and thirty-two underwent FS-LASIK. Corneal fluorescein (FL) staining, tear break-up time (TBUT), Schirmer I test (SIT), ocular surface disease index (OSDI) and central corneal sensitivity were evaluated in all participants. Tears were collected and analyzed for interleukin-6 (IL-6), tumor necrosis factor-α (TNF-α), nerve growth factor (NGF) and intercellular adhesion molecule-1 (ICAM-1) levels using multiplex magnetic beads. All measurements were preformed preoperatively and 1 day, 1 week, 1 month and 3 months postoperatively.

**Results:**

FL scores in ReLEx smile group were lower than those of FS-LASIK group 1 week postoperatively (P = 0.010). Compared to the FS-LASIK group, longer TBUT were observed in ReLEx smile group 1 month (P = 0.029) and 3 months (P = 0.045) postoperatively. No significant differences were found in tear secretion for the two groups (P>0.05). OSDI scores were higher in FS-LASIK group 1 month after surgery (P = 0.020). Higher central corneal sensitivity was observed in ReLEx smile group 1 week, 1 month and 3 months (P<0.05) postoperatively. Compared to FS-LASIK group, lower and faster recovery of IL-6 and NGF levels in tears was observed in ReLEx smile group postoperatively (P<0.05). Tears TNF-α and ICAM-1 concentrations were not significantly different between the two groups at any follow-up time (P>0.05). Moreover, IL-6 and NGF levels correlated with ocular surface changes after ReLEx smile or FS-LASIK.

**Conclusions:**

In the early postoperative period, ReLEx smile results in milder ocular surface changes than FS-LASIK. Furthermore, the tear inflammatory mediators IL-6 and NGF may play a crucial role in the ocular surface healing process following ReLEx smile and FS-LASIK.

## Introduction

The femtosecond laser, which is characterized by its outstanding cutting precision and less-severe complications, has been widely applied in laser-assisted in situ keratomileusis (LASIK) [1]–[3]. This technique, known as femtosecond laser-assisted laser in situ keratomileusis (FS-LASIK), involves flap creation and stromal ablation using femtosecond laser and excimer laser, respectively. Recently, refractive lenticule extraction (ReLEx), which uses exclusively the femtosecond laser, has become a novel alternative to refractive surgeries [4]. ReLEx, which can be further divided into femtosecond lenticule extraction (FLEx) and small-incision lenticule extraction (SMILE) based on the method by which the lenticule is removed, has proven to be an effective procedure in visual clinical outcomes [4]–[9].

Ocular surface damage due to flap formation and stromal ablation is associated with post-LASIK dry-eye and other rare complications such as epithelial ingrowth and myopia regression [10]–[14]. Post-LASIK patients often experience dry eye symptoms, which are usually transient and manageable using topical medications. The mechanisms underlying postoperative dry eye have not been well clarified. A large number of studies conclude that LASIK and other ablation refractive surgeries caused corneal nerve damage, contributing to the development of dry eye [15]–[17]. Ocular surface damages occurring during refractive surgeries also stimulate low or mild inflammation and increase the levels of tear inflammatory mediators (including cytokines, chemokines and growth factors) during early postoperative period. Epithelial cells and keratocytes express the receptors for tear inflammatory mediators, which are proposed to be involved in the corneal wound-healing process and responsible for post-LASIK complications [18]–[20].

ReLEx, which uses only a femtosecond laser to make a very precise corneal cut to create an intact piece of intrastromal lenticule matching the patient's refractive error, marks a milestone in laser refractive surgeries. It is superior to LASIK in terms of accuracy, reversibility and patient comfort. ReLEx can be further divided into ReLEx flex and, a less invasive procedure, ReLEx smile. ReLEx flex, similar to FS-LASIK, necessitates the creation of a flap while ReLEx smile requires only a small-incision to remove intrastomal lenticule [21], [22]. Therefore, in theory, ReLEx smile should have milder influences on the ocular surface compared to FS-LASIK. A recent animal study by Dong et al. reported less keratocyte apoptosis, proliferation and inflammation in ReLEx smile group compared with FS-LASIK group [23]. Moreover, a nonrandomized clinical study found milder dry eye symptoms in ReLEx smile versus FS-LASIK patients [24]. However, to the best of our knowledge, there are no previous human studies comparing ocular surface inflammatory responses after ReLEx smile and FS-LASIK. Thus, the aim of the present study is to investigate ocular surface changes with an especial focus on determining any effects of tear inflammatory mediators on myopic subjects after ReLEx smile and FS-LASIK.

## Materials and Methods

### Subjects

This prospective study recruited 47 patients who underwent either bilateral ReLEx smile (n = 15) or FS-LASIK surgeries (n = 32) for the treatment of myopia at Zhongshan Ophthalmic Center (Guangzhou, China) between June 2012 and January 2013. Participants had a refractive error ranging from −3.00 to −8.00 diopters (D), with or without astigmatism. Patients were eligible for inclusion in the initial study if they were 18 years of age or older, had a monocular best corrected visual acuity of 20/20 or better and had stable refractive errors (less than 0.5D) over the preceding 2 years. The patients with any of the following conditions were excluded from our studies: patients with diabetes mellitus, systemic collagen vascular disease, corneal disease, glaucoma and a history of ocular disease, usage of tear supplements or contact lens wearing during the past year.

All the patients completed four postoperative follow-up visits. One eye of each patient was randomly chosen for statistical analysis. A single surgeon performed all of the FS-LASIK and ReLEx smile surgeries following standard procedures. All subjects provided written consent to participate in this study. All procedures were approved by the Ethics Committee of Zhongshan Ophthalmic Center of Sun Yat-sen University in compliance with the tenets of the Declaration of Helsinki and obtained ethics.

### SMILE Procedure

ReLEx smile surgery was performed using the VisuMax femtosecond laser system (Carl Zeiss Meditec AG, Jena, Germany) with a 500 kHz repetition rate. Four fundamental cleavage planes were sequentially created as follows: (1) the posterior surface of the refractive lenticule (spiral in); (2) the 360°cordal length vertical edge of the refractive lenticule; (3) the anterior surface of the refractive lenticule (spiral out); (4) a small side-cut incision. Once the four cleavage planes were created, suction was released automatically and the anterior and posterior surfaces of refractive lenticule were separated from the overlying cornea and underlying stroma respectively using a thin blunt spatula. The targeted free lenticule was then grasped with a Blum forcep and extracted from the small side-cut incision. For all ReLEx smile procedures, the optical zone size was 6.0 mm and the femtosecond laser used to create the four cleavage planes has an energy of 140 nJ. The anterior lenticule surface was 110 µm deep. The small incision was located in the 120°position, with 50°cordal length (the side-cut incision with a circumferential length of 4.0–5.0 mm and angle of 90°). The spot spacing and tracking spacing were 3.0 µm for the lenticule, 2.5 µm for the lenticule side cut, 3.0 µm for the small incision and 2.0 µm for the small incision side cut. The entire suction time was 39–40 seconds in all cases.

### FS-LASIK Procedure

The FS-LASIK procedure was performed using the VisuMax femtosecond laser system (Carl Zeiss Meditec AG, Jena, Germany) for flap creation and the WaveLight Allegretto excimer laser system (WaveLight GmbH, Germany) for stromal ablation. The femtosecond laser used for flap creation has an energy of 140 nJ with a 500 kHz repetition rate. The flap had a diameter of 8.0 mm, thickness of 110 µm and standard superior hinge with a 50°angle. The track and spot spacing were 3.0 µm for flap creation and 1.5 µm during flap side-cutting. After stromal ablation, the flap was carefully repositioned.

Postoperatively, patients in both ReLEx smile and FS-LASK groups received tobramycin/dexamethasone eyedrops (Tobradex, Alcon), 0.5% levofloxacin eyedrops (Cravit, Santen) and carboxxymethylcellulose sodium eyedrops (Allergan) four times a day for one week. Tobradex and Cravit were then suspended, while artificial tear supplements were prescribed for another three weeks.

### Ocular Surface Evaluation

Before surgery and 1 day, 1 week, 1 month, 3 months postoperatively, the following ocular surface parameters were evaluated on all participants as previously described [25]: corneal fluorescein (FL) staining, tear break-up time (TBUT), Schirmer I test (SIT), ocular surface disease index (OSDI) and central corneal sensitivity. OSDI, a 12-question survey, is one of the most widely used tools for dry eye patients to report their symptoms. Each question is given a rank between 0–4. OSDI score  =  (sum of score ×25)/(number of questions answered), ranging from 0–100 score. Central corneal sensitivity was measured with a Cochet-Bonnet esthesiometry (Luneau Ophthalmlogie, Chartres, France). This test was not performed on patients on the first day after surgery as the procedure can potentially injure the cornea. A single observer performed preoperative and postoperative ocular surface evaluations.

### Tear Sample Collection

Before surgery and 1 day, 1 week, 1 month postoperatively, 10 µl basal tear samples were collected in disposable 2 µl microcapillaries (microcaps 2 µl,Drummond Scientific Co, USA) from the inferior meniscus without anesthesia. Care was taken to avoid touching the ocular surface or lid margin. In order to exclude the dilution effect caused by hypersecretion, the tear flow rate was carefully controlled. Collected tears were immediately transferred into 0.5 ml microtubes (Eppendorf) and stored at −80°C until use. For the determination of inflammatory mediators, each tear sample was diluted 1∶5 with assay diluents and 50 µl of each sample was used for the subsequent assays.

### Assays for Inflammatory Mediators Using Bio-plex System

Tears were assayed for interleukin-6 (IL-6), tumor necrosis factor-α (TNF-α), nerve growth factor (NGF) and intercellular adhesion molecule-1 (ICAM-1). The concentrations of these inflammatory mediators were determined by multiplex magnetic bead technology using commercially available quantitative kits (Procarta; Affmetrix Inc, Santa Clara, CA, USA) following the manufacturer's instructions. The final results were adjusted by the dilution factor. To avoid intential or unconscious bias and ensure consistent evaluation, all assays were performed blind by a single technician.

### Statistical Analysis

Statistical analysis was performed using an SPSS software package (version 13.0 SPSS, Chicago, IL, USA). Comparisons between the two treatment groups, including OSDIs and inflammatory mediators concentrations, were performed by applying two-tailed Student's t-test (normally distributed data) or Wilcoxon rank sum test (non-normally distributed data). Compared with preoperative level, an ANOVA for repeated measurements was applied to analyze the data. For TBUT, SIT, central corneal sensitivity and tear cytokines, the significant level was 0.05(LSD-t). But for corneal FL staining and OSDIs, the adjusted level was established at 0.017 (Bonferroni). Pearson product-moment or Spearman rank correlation was used to check for correlations between tear inflammatory mediators and ocular surface parameters with the significant level of 0.05.

## Results

The preoperative characteristics of the study population are shown in table 1. Patients in ReLEx smile and FS-LASIK groups were matched in terms of age, initial uncorrected visual acuity, best spectacle-corrected visual acuity, mean spherical equivalent, central corneal thickness and corneal curvature (P>0.05).

**Table 1:** Preoperative descriptive statistics of subjects (Mean±SD).

Group	ReLEx smile	FS-LASIK	P-value
Ages (years)	24.53±4.05	22.75±4.28	0.130
Uncorrected visual acuity (logMAR)	0.96±0.11	0.95±0.15	0.513
Best spectacle-corrected visual acuity (logMAR)	−0.12±0.08	−012±0.09	0.659
Spherical equivalent (D)	5.65±1.18	5.22±1.77	0.401
Central corneal thickness (um)	543.60±25.43	539.72±23.61	0.611
Corneal curvature (D)	43.51±1.09	43.77±1.13	0.240

### Ocular Surface Parameters

No significant differences were found between the two treatment groups for the preoperative ocular surface parameters, including corneal FL staining, tear stability, tear secretion, OSDI scores and central corneal sensitivity (P>0.05).

**Figure 1:**
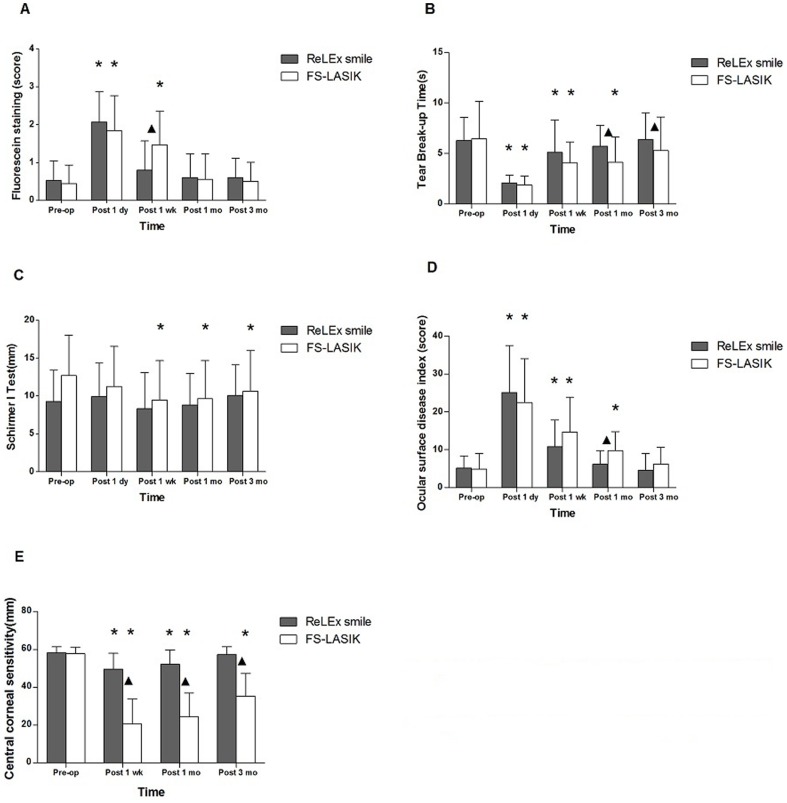
Ocular surface parameters after ReLEx smile and FS-LASIK. Ocular surface parameters, including Corneal fluorescein (FL) staining, tear break-up time (TBUT), Schirmer I test (SIT), ocular surface disease index (OSDI) and central corneal sensitivity, were compared between patients undergoing ReLEx smile and FS-LASIK. ▴: significant differences between ReLEx smile and FS-LASIK. *: significant differences compared with preoperative level.

For corneal FL staining, the scores in both ReLEx smile and FS-LASIK groups increased significantly at postoperative 1 day compared with preoperative values (P<0.017). The scores in ReLEx smile group had recovered to preoperative levels by 1 week after surgery, while the scores were still higher in FS-LASIK group until 1 month postoperatively (P>0.017). ReLEx smile patients had lower corneal FL staining scores than FS-LASIK patients at 1 week postoperatively (P = 0.010), but there was no significant difference in staining score at other follow-up time points between the two groups (P>0.05). (Figure 1,A).

The stability of the tear film was assessed by TBUT measurement. The TBUT in both treatment groups was significantly shortened 1 day and 1 week postoperatively compared with preoperative measurements. Similar to corneal FL staining, a quicker TBUT recovery was observed in ReLEx smile (1 month) than in FS-LASIK patients (3 month). The tear stability of ReLEx smile patients was found to be superior to that of FS-LASIK patients at 1 month (P = 0.029) and 3 months (P = 0.045) postoperatively, but no differences were observed at 1 day and 1 week (P>0.05) postoperatively. (Figure 1,B)

In comparison with preoperative level, the tear secretions in ReLEx smile patients were not significantly changed after surgery (P>0.05), while a subtle, but significant reduction of tear secretions was observed for FS-LASIK patients 1 week, 1 month and 3 months postoperatively (P<0.05). However, tear secretions of two groups were not significantly different at any follow-up time points (P>0.05). (Figure 1,C)

Dry eye symptoms were assessed by OSDI questionnaires. Consistent with quicker recoveries of corneal FL staining, tear film stability and tear secretion in ReLEx smile patients, we found that the OSDI scores of ReLEx smile and FS-LASIK group reached preoperative levels by postoperative 1 month and 3 months, respectively (P>0.017). The OSDI scores of ReLEx smile group were lower than those of FS-LASIK group 1 month after surgery (P = 0.020). There were no significant differences in OSDI scores between the two groups at other follow-up times. (Figure 1,D)

The central corneal sensitivity of ReLEx smile patients was only slightly decreased after surgery (P<0.05) and reached to preoperative level 3 month postoperatively (P>0.05). However, a robust reduction in central corneal sensitivity was observed in patients undergoing FS-LASIK, and the central corneal sensation in FS-LASIK patients was still significantly lower than preoperative level at the end of our three-month follow-up period (P<0.05). Moreover, the central corneal sensitivity of FS-LASIK group was significant lower in comparison with that of ReLEx smile group 1 week, 1 month and 3 months postoperatively (P<0.05). (Figure 1,E)

### Tear Inflammatory Mediators

**Figure 2:**
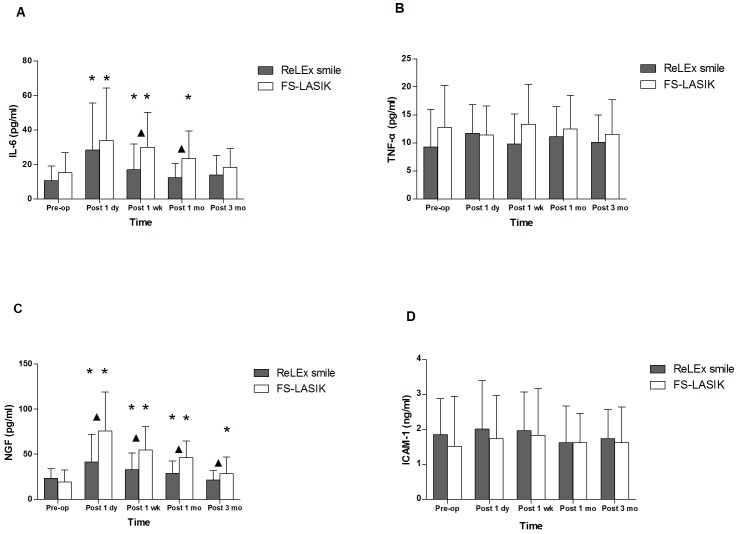
The concentrations of inflammatory mediators in tears after ReLEx smile and FS-LASIK. The tear levels of inflammatory mediators including interleukin-6 (IL-6), tumor necrosis factor-α (TNF-α), nerve growth factor (NGF) and intercellular adhesion molecule-1 (ICAM-1) was evaluated in patients undergoing ReLEx smile and FS-LASIK ▴: significant differences between ReLEx smile and FS-LASIK. *: significant differences compared with preoperative level.

In this study, four inflammatory mediators were examined: IL-6, TNF-α, NGF and ICAM-1. No statistical differences were found between the ReLEx smile and FS-LASIK groups for the preoperative tear levels of these inflammatory mediators (P>0.05). (Figure 2)

Whereas both ReLEx smile and FS-LASIK procedures resulted in an increase in IL-6 tear levels (P<0.05), the ReLEx smile group recovered faster, reaching the preoperative level of IL-6 at 1 month after surgery in comparison with 3 months after surgery for FS-LASIK group. Tear IL-6 concentrations were lower in the ReLEx smile group than that in the FS-LASIK group at 1 week (P = 0.015) and 1 month (P = 0.006) postoperatively. (Figure 2A)

A similar pattern of changes was observed for NGF concentrations. Tear NGF levels increased after both ReLEx smile and FS-LASIK surgeries. However, NGF levels in the ReLEx smile group recovered to the preoperative level by 3 months after surgery (P>0.05), while NGF levels in the FS-LASIK group was still slightly higher at the end of our 3-month follow-up period compared with preoperative NGF levels. Moreover, the tear NGF concentrations were also lower in ReLEx smile group compared with the FS-LASIK group at any follow-up visit (P<0.05). (Figure 2B)

At any postoperative follow-up time, there were no significant differences in the levels of TNF-α and ICAM-1 before or after surgery in either group (P>0.05). Moreover, tears TNF-α and ICAM-1 levels did not differ significantly between the two treatment groups (P>0.05). (Figure 2)

### Correlations between Inflammatory Mediators and Ocular Surface Changes

IL-6 levels were positively correlated with OSDI scores (r = 0.363, P = 0.004) in the ReLEx smile group, and FL scores (r = 0.215, P = 0.015) and OSDI scores (r = 0.200, P = 0.023) in the FS-LASIK group. There were positive correlations between FL scores (r = 0.249, P = 0.005), OSDI scores (r = 0.277, P = 0.002) and NGF levels in the FS-LASIK group. Negative correlations between NGF levels and central corneal sensitivity were found in both ReLEx smile (r = −0.335, P = 0.024) and FS-LASIK groups (r = −0.296, P = 0.003).

## Discussion

Ocular surface damage contributes to the development of noninfective post-LASIK complications including dry eye and other unsatisfactory sequelae. The evolutionary direction of refractive surgery is to develop less invasive surgical procedures. ReLEx smile is a novel refractive surgery, which uses the femtosecond laser system as an all-in-one device for lenticule processing and substitutes small incision for corneal flap. Therefore, healthier ocular surface in ReLEx smile patients is expected, compared to patients undergoing FS-LASIK or conventional refractive surgeries. In the current study, we systematically compared early ocular surface changes and inflammatory response after ReLEx smile and FS-LASIK.

In the present study, we observed similar ocular surface changes in both ReLEx smile and FS-LASIK patients 1 day after surgery, including tear film stability, tear secretions, central corneal sensitivity, and an increase in corneal fluorescein staining and OSDI scores. Although ReLEx smile surgery avoids flap creation, more severe damage to tissue surrounding the small incision is still probably involved, because of the dissociation and extraction of stromal lenticule. Moreover, we speculated that the high variation of ocular surface in patients 1 day postoperatively might also contribute to the minor differences between two surgical procedures.

A previous study has reported that the ocular surface damage related to FS-LASIK was milder than that after conventional LASIK due to the creation of a thinner flap and the application of lower suction pressure [2]. FS-LASIK still involves flap creation, while ReLEx smile substitutes small incision for corneal flap. Thus, milder ocular surface damage would be expected in ReLEx smile patients. In our study, evaluation of ocular surface damage did show a faster recovery of all corneal surface parameters to preoperative level in ReLEx smile compared to FS-LASIK patients. In addition, more favorable outcomes of FL staining, TBUT, OSDI and central corneal sensitivity were also observed in ReLEx smile compared to that in FS-LASIK patients at certain follow-up time points. Consistent with our data, another study group recently reported that ReLEx smile was superior to FS-LASIK in terms of dry eye symptoms and corneal sensation [24].

The possible mechanisms underlying the significant advances in ReLEx smile are as follows: To begin with, as a flapless procedure, ReLEx smile may reduce the number of corneal nerve fibers affected during the surgeries thus alleviating dry eye symptoms and signs. The alteration of corneal innervation after LASIK is reported to be one of the most likely causes contributing to postoperative dry eye [26]. Loss of corneal innervation during flap creation results in corneal and conjunctival hypersensitivity to ocular dryness, which is known as “LASIK-induced neuropathic dry eye” [27]. Moreover, corneal nerve damage might affect the reflex loops between the cornea-blink and cornea-lacrimal gland, leading to reduction in tear secretion and tear film stability [12]. Nerve bundles enter the cornea from the periphery towards the center in a radial fashion parallel to the corneal surface [28]. Meanwhile, the majority of stromal nerve fibers run through the anterior third of the corneal stroma and innervate the corneal epithelium forming the epithelial nerve network [29]. The characteristics of corneal nerve innervation were supportive of our results. The involvement of small incision in ReLEx smile procedure should have reduced the effects on corneal nerve fibers located in the anterior stroma as well as the peripheral corneal epithelium. Our data revealed a faster recovery of ocular surface damages in ReLEx smile than in FS-LASIK group, which should be mainly attributed to the milder corneal nerve impairment during this new flapless technique. In agreement with our results, a recent study reported that the decrease in corneal subbasal nerve fiber density was more severe in FS-LASIK compared with ReLEx smile patients during early postoperative period [30].

What's more, corneal surface topography also contributes to tear film stability. We speculated that the flapless ReLEx smile procedure offers a more regular corneal surface and therefore leads to a faster recovery of tear film stability when compared with FS-LASIK, which involves corneal flap creation. However, further investigations are needed to test this hypothesis.

Refractive surgeries induce low to mild inflammatory response, which have been proven to be involved in the postoperative wound-healing process [18]–[20]. No previous study has compared the inflammatory response between ReLEx smile and FS-LASIK. In this study, we observed an upregulation of IL-6 levels in tears after ReLEx smile as well as FS-LASIK, with higher IL-6 levels and slower recovery to preoperative levels in FS-LASIK patients. As a constant component of the normal human tear fluid, IL-6 is a proinflammatory cytokine synthesized by keratocytes and endothelial cells [31]. Previous studies suggested that IL-6 might play an important role in several sterile ocular surface inflammation and was also known to be involved in promoting corneal wound healing [32], [33]. Moreover, elevated levels of IL-6 have been observed in the tears of patients suffering from dry eye, ocular chemical injury and contact lenses wear [34]–[36]. In our study, correlation analysis suggested that the increase in IL-6 concentrations after these two recent refractive surgeries might be associated with damages to the ocular surface resulting from the laser techniques. The relatively milder ocular surface damage caused by the ReLEx smile technology was consistent with lower IL-6 levels after ReLEx smile compared to FS-LASIK.

In the present study, we found the same pattern of dynamic changes in NGF levels as in IL-6 after ReLEx smile and FS-LASIK. NGF is primarily expressed by epithelial and stromal cells, which populate both conjunctiva and cornea over the ocular surface. Recently, NGF was found to accelerate epithelium healing, induce keratocyte migration and facilitate corneal nerve regeneration [37], [38]. NGF release is activated by the signaling cascade during corneal healing process. Several proinflammatory cytokines excreted by injured corneal epithelial cells and stromal keratocytes can upregulate the expression of NGF and its receptors [39]. Lee HK et al. reported that the concentrations of NGF increased to varying levels after photorefractive keratectomy (PRK) and conventional LASIK [40]. Our study further found that postoperative NGF concentrations in tears were higher in FS-LASIK group when compared with the ReLEx smile group. Moreover, correlation analysis indicated that the discrepancy of NGF levels between these two surgeries might be also associated with the severity of damage to the cornea surface. In this new smile procedure, the lenticule dissection substitutes stromal ablation and small incision replaces flap formation. Therefore, we speculated that the minor dysfunction of ocular surface induced by smile surgery failed to stimulate NGF expression.

Another possible factor affecting the concentrations of IL-6 and NFG in tears could be the alteration of tear secretion. In our study, care was taken to control the flow rate while collecting tear samples, to exclude the possibility of hypersecretion. Moreover, the preoperative and postoperative tear secretion was comparable, as assessed via Schirmer I test. There were no significant differences in tear secretion between ReLEx smile and FS-LASIK patients before or after the surgeries. Therefore, tear secretion might not be the reason contributing to the dynamic changes in IL-6 and NFG postoperative concentrations. Taken together, IL-6 and NGF can function as biomarkers for ocular surface damages after refractive surgeries.

As a proinflammatory cytokine, TNF-α is expressed by corneal epithelium and inflammatory cells, which are also considered to participate in the corneal wound healing [41]. It has been reported that TNF-α levels are elevated in tears of patients with dry eye syndrome [42]. Surprisingly, our study did not show any significant changes in TNF-α concentrations before and after ReLEx smile and FS-LASIK. Vesaluoma M, et al. found that, even after PRK, merely a transient and slight upregulation in the TNF-α levels was observed [43]. Accordingly, in our study, it would be most likely that the mild ocular surface changes after these two recent surgeries might not be great enough to induce detectable increase in TNF-α levels. ICAM-1 is a conserved member of the immunoglobulin supergene family, functioning as an adhesion molecule [44] and in corneal wound healing [45]. Similarly, no significant changes in ICAM-1 levels were observed after either of the two surgeries in our study. It is quite possible that the reasons for this tendency were similar to that of TNF-α. Further investigations are required to verify these speculations.

Finally, there are some limitations in the present study. For example, we did not employ randomized design, even though the patients in both treatment groups were matched in terms of age, refractive state, and ocular surface health. In addition, only four inflammatory mediators were determined in our study, and more cytokines are still required to be analyzed in the future. Moreover, long-term characteristics of ocular surface and tear inflammatory mediators are also worth investigating in further research.

In conclusion, this prospective study demonstrated that ReLEx smile results in milder effects on ocular surface compared with FS-LASIK at early postoperative period. IL-6 and NGF might play a crucial role in the ocular surface healing process after ReLEx smile and FS-LASIK. Thus, the molecular mechanisms underlying the ocular surface wound healing should be further investigated.
